# Copy Number Variation‐Based Molecular Sexing of *Ixodes scapularis* and *Rhipicephalus microplus* Immature Stages Using qPCR and ddPCR Approaches

**DOI:** 10.1002/ece3.74562

**Published:** 2026-10-28

**Authors:** Arvind Sharma, Benjamin Faustino, Isaac Hinne, Jakub Wudarski, Saransh Beniwal, Nurul Hamidi, Kyla Ercit, Sanjay Basu, Malimba Lisulo, Mattia Poletto, Kelly J. Matzen, Monika Gulia‐Nuss

**Affiliations:** ^1^ Department of Biochemistry, Molecular Biology, and Biotechnology University of Nevada Reno Nevada USA; ^2^ Oxitec Ltd Abingdon Oxfordshire UK; ^3^ Division of Immunology, The Roslin Institute University of Edinburgh, Easter Bush Campus Midlothian UK

## Abstract

Accurate sex identification of immature ticks is essential for understanding sex‐specific ecological dynamics, pathogen transmission, and reproductive biology. However, tick larvae and nymphs lack morphological sexual dimorphisms, limiting such studies. Here, we report the development and validation of a copy number variation (CNV)‐based molecular sexing approach for two important hard tick species: *Ixodes scapularis* , a major public health vector of human pathogens, and *Rhipicephalus microplus* , an ectoparasite responsible for significant economic losses in the cattle industry. Using newly published chromosomal‐level genome assemblies and whole‐genome resequencing data, we identified female‐enriched CNVs in two genes—calcium/calmodulin‐dependent 3′,5′‐cyclic nucleotide phosphodiesterase 1A‐like (*NPD*) and rap guanine nucleotide exchange factor 2‐like (*RAPGEF2*)—and developed SYBR‐Green and probe‐based qPCR and droplet digital PCR (ddPCR) assays using DNA extracted non‐destructively from a single leg, thus preserving ticks for continued feeding, molting, and behavioral analysis. In *I. scapularis* , we were able to assign the sex of 91.3% of nymphs based on results from at least two molecular tests, and these assignments were confirmed when the nymphs later molted into adults. Similarly, in *R. microplus* , the qPCR assay revealed clear, sex‐linked differences in gene copy number. Together, these findings indicate that CNV‐based markers are a reliable and widely applicable method for sex determination in immature ticks.

## Graphical abstract

We developed and validated a copy number variation (CNV)‐based molecular method to accurately determine the sex of immature ticks, *Ixodes scapularis* and *Rhipicephalus microplus* , which lack morphological sexual dimorphism. Using qPCR and ddPCR assays targeting female‐enriched genes, sex was reliably identified from DNA extracted non‐destructively from a single leg, with validation through adult emergence. This minimally invasive approach enables robust investigation of sex‐specific tick biology, vector competence, and population dynamics.

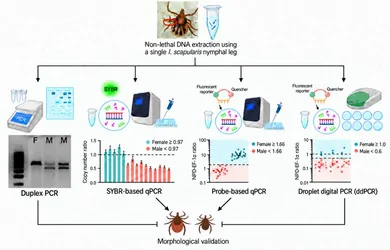

## 1 Background

The increasing prevalence of Lyme disease (LD) and the emergence of other tick‐borne human diseases have become a major public health concern worldwide. Ticks are vectors of numerous viruses, bacteria, and protozoa that can impact humans, companion animals, and domestic livestock (Jongejan and Uilenberg 2004). Blacklegged tick, *Ixodes scapularis* (Ixodida: Ixodidae), is the primary vector of at least seven pathogens that cause human diseases, including *Borrelia burgdorferi* , the causative agent of LD; *Babesia microti* (human babesiosis), *Anaplasma phagocytophilum* (human granulocytic anaplasmosis); *Borrelia miyamotoi* (hard‐tick relapsing fever), *B. mayonii* (new Lyme *Borrelia* spp.), *Ehrlichia muris* ‐like agent (ehrlichiosis); and Powassan virus (Powassan virus disease) (reviewed in Stafford et al. (2017)). Since LD was first described in the 1970s, the number of reported human cases in the United States has steadily increased, largely due to the range expansion of *I. scapularis* and the spread of *B. burgdorferi* (Diuk‐Wasser et al. 2012; Eisen et al. 2016; Kugeler et al. 2015; Pepin et al. 2012). The actual incidence of human disease cases is estimated to be at least 10‐fold greater than reported confirmed cases, at ≈450,000 annually (Kugeler et al. 2015).

While *I. scapularis* and LD have been the impetus for recent research on tick ecology and management, the Asian blue tick, *Rhipicephalus microplus* , has a widespread distribution in many tropical and subtropical areas of Asia, South and Central America, and most of sub‐Saharan Africa (Angus 1996; Bermúdez et al. 2022; Guglielmone et al. 2006; Kanduma et al. 2020; Tan et al. 2021). It is a one‐host tick that primarily infests cattle and is one of the most detrimental vectors of veterinary pathogens, such as *Babesia bovis*, *Babesia bigemina*, and *Anaplasma marginale* , costing the worldwide agriculture industry billions of dollars a year (Bock et al. 2004; Lew‐Tabor and Rodriguez Valle 2016; Todorovic et al. 1981). The spread of *R. microplus* to new areas in the tropics is a significant threat to livestock industries and the livelihoods of rural populations, who often depend on livestock for their survival. The economic importance of *R. microplus* is also compounded by its propensity to displace other native tick species, its higher vectoral capacity, and its ability to develop resistance to acaricides (Abbas et al. 2014; Dzemo et al. 2022; Nyangiwe et al. 2013). Although *R. microplus* has been eradicated from the USA, the threat remains due to cattle exports and the risk of ticks reinfesting from nearby countries through trade (Busch et al. 2014).

The transmission of tick‐borne pathogens in natural settings primarily relies on the larval and nymphal stages of ticks, which commonly feed on the same vertebrate reservoir hosts (Piesman and Gern 2004). Although these early stages are key to pathogen spread, the specific roles of male and female immature ticks remain mostly unknown. One major obstacle in understanding these dynamics is the absence of morphological differences during immature stages. Unlike adults, which can be sexed based on morphological traits, immature ticks are visually indistinguishable, and current sexing methods are often invasive, require specialized equipment, or are only applicable after molting (Dusbabek 1996; Hu and Rowley 2000; Keirans et al. 1996).

Molecular sexing methods have been developed for various arthropod species, often utilizing sex‐specific DNA sequences to distinguish between males and females. This approach has been successfully applied to organisms such as the silkworm *Bombyx mori* (Abe et al. 1998), the malaria mosquito *Anopheles gambiae* (Krzywinski et al. 2004), and more recently, the black legged tick *I. scapularis* (Ronai et al. 2025). However, current tick‐specific methods are in the initial stages of development and have not been further adapted into high‐sensitivity methods, such as real‐time quantitative PCR (qPCR) or droplet digital PCR (ddPCR), which would facilitate broader application in research and tick colony management. Additionally, the only published method to date is for *I. scapularis* and has not been tested on other tick species.

Ixodidae, the hard ticks, are divided into two subfamilies: Prostriata and Metastriata. The Prostriata contain the genus Ixodes (including *I. scapularis* ) and have an XX:XY sex determining system (Gulia‐Nuss et al. 2016; Meyer et al. 2010; Nuss et al. 2023). In the Metastriata, most species have lost the Y chromosome and have an XX:XO sex‐determining system (Chensheng et al. 1994; Oliver 1977). *Rhipicephalus microplus* is a metastriate tick with an XX:XO sex‐determining system (Newton et al. 1972; Oliver and Bremner 1968). These differences in sex determination suggest that copy number variation (CNV) in sex‐linked genes may enable an assay applicable to multiple species. Similar CNV‐based approaches have been developed for other arthropods, such as in *Leptinotarsa decemlineata* , the Colorado potato beetle, where sex was successfully identified across different life stages using CNV (Sedláková et al. 2022).

Developing a molecular tool to rapidly and accurately identify male and female ticks at immature life stages is valuable for studying their roles in pathogen transmission (Koloski, Adam, et al. 2025; Koloski, Sharma, et al. 2025) and for developing genetic control strategies. Genetic control strategies, including CRISPR‐Cas9‐based gene drives, control via self‐limiting genetic systems (Spinner et al. 2022), and sterile male techniques, have been successfully applied in mosquitoes; however, these approaches remain unexplored in ticks (Feng et al. 2021; Kyrou et al. 2018; Wang et al. 2021), primarily due to limited biological and technical knowledge, including the absence of molecular sexing tools. Developing a reliable method to determine tick sex at immature stages will be essential for adapting the genetic control strategies that have proven successful in insect research.

In this study, we developed a molecular sex identification assay using CNVs identified from the recently published chromosome‐level genome assemblies of *I. scapularis* and *R. microplus* (Deng et al. 2024; Mou et al. 2025; Nuss et al. 2023; Tidwell et al. 2024). Through bioinformatics approaches, we identified a highly variant gene encoding calcium/calmodulin‐dependent 3′,5′‐cyclic nucleotide phosphodiesterase 1A‐like (*NPD*). We compared three approaches for *I. scapularis* sex determination: SYBR Green‐based qPCR, fluorescent probe‐based qPCR, and droplet digital PCR (ddPCR) using DNA from a single leg, thus keeping the nymphs intact for feeding and molting. We compared our methods with the published duplex PCR assay for *I. scapularis* (Ronai et al. 2025). After testing with molecular methods, nymphs were blood‐fed and allowed to molt, allowing morphological confirmation. We further confirmed *NPD* and an additional CNV gene, rap guanine nucleotide exchange factor 2‐like (*RAPGEF2*), in *R. microplus* ticks using a probe‐based qPCR assay and whole nymph DNA. This approach offers a flexible and high‐confidence method for sexing nymphal ticks, a life stage where visual differentiation is not possible, and shows its applicability in more than one species.

## 2 Methods

### 2.1 Ticks Rearing and Feeding

#### 2.1.1 *Ixodes scapularis*

Pathogen‐free nymphs and adults were purchased from the Oklahoma State University (OSU) tick rearing facility and kept in an incubator at 95% relative humidity and 20°C in our laboratory (Nuss et al. 2017). Nymphs were blood‐fed on the dorsal surface of a New Zealand white rabbit (Jackson Laboratories), which was shaved one day before feeding. Based on the putative sex, determined using molecular methods, nymphs were divided into three groups: male, female, and undetermined. Each group was placed in a separate feeding chamber (3‐in. plastic capsules) affixed to the shaved skin using Kamar adhesive (Levin et al. 2014; Reyes et al. 2024) and was allowed to feed to repletion. After feeding, individual nymphs were transferred to 1.5 mL tubes and stored at 20°C and 95% relative humidity for recovery and molting. All procedures were approved by the Institutional Animal Care and Use Committee (IACUC) at the University of Nevada, Reno (IACUC # 21‐01‐1118).

#### 2.1.2 *Rhipicephalus microplus*

Pathogen‐free ticks from the La Minita *R. microplus* strain were sourced from the USDA‐ARS (Moscow, ID, USA) and maintained at the Large Animal Research and Imaging Facility, The Roslin Institute (University of Edinburgh, U.K.). Larvae (3–4 months old) were placed on female Holstein‐Friesian calves sourced from the University of Edinburgh, where they fed and developed through the larval and nymphal stages before molting into adults on the host. Engorged adult female ticks were maintained in 50 mL tubes at 28°C and 80%–90% relative humidity and allowed to lay eggs to recover immature life stages. All procedures were conducted in full compliance with the Animal (Scientific Procedures) Act 1986 (revised 2013). Unfed *R. microplus* adult ticks of known sex, determined by morphological examination, were used to identify variable genes and to develop the sexing assay and were later included as reference controls to validate the assay. Unfed adults were obtained by removing encased nymphs from the host 12 days post‐infestation. Nymphs were allowed to molt at 28°C and 90% RH for two days. Unfed nymphs were collected five days post‐infestation as encased larvae and allowed to molt off‐host at 28°C and 90% RH.

### 2.2 DNA Extraction and Quantification

#### 2.2.1 *Ixodes scapularis*

The genomic DNA from unfed nymphs and adults was extracted from a single leg by cutting the patella segment of the third leg pair under a dissecting microscope. gDNA was extracted using a BioSearch QuickExtract DNA extraction buffer (Biosearch Technologies #QE09050) following the manufacturer's protocol. Clipped legs were placed in extraction buffer and vortexed for 15 s, followed by incubation at 65°C for 6 min and 98°C for 2 min. The solution was vortexed for 15 s between incubations. The concentration of extracted gDNA was estimated by using a Nanodrop spectrophotometer, and the quality of the gDNA was assessed by PCR amplifying a housekeeping gene (*β‐actin*). The gDNA was stored at −20°C.

#### 2.2.2 *Rhipicephalus microplus*

Genomic DNA was extracted using the Qiagen DNeasy Blood and Tissue Kit (Qiagen, Venlo, Netherlands), with the following changes made to the supplementary insect protocol: (1) whole ticks were homogenized with a pestle in 10 μL of RL buffer taken from the Norgen Total RNA Purification Kit (Norgen Biotek, Thorold ON, Canada); (2) after the addition of buffer AL and before the addition of ethanol, a second incubation step of 10 min at 70°C was added; (3) a second elution step was added where the eluate was pipetted back onto the column and spun again. Eluted DNA was quantified using a NanoPhotometer.

### 2.3 Gene Selection

#### 2.3.1 *Ixodes scapularis*

PacBio HiFi reads from one male and two female *I. scapularis* samples were adapter‐filtered and aligned to the reference genome (Nuss et al. 2023). Mean coverage was calculated across non‐overlapping 2 kb windows and normalized using median autosomal coverage. Female‐to‐male coverage ratios were calculated for each female sample, and sex‐biased regions showing consistent patterns across samples and spanning consecutive windows were merged. Genes overlapping these regions were identified using the corresponding annotation file.

Eight candidate genes showing sex‐specific CNVs were identified from this analysis (Table S1), and all were mapped to the recently published chromosome‐level genome of *I. scapularis* (IscapMGN; GCA_031841145.1) (Nuss et al. 2023). Out of the top eight, calcium/calmodulin‐dependent 3′,5′‐cyclic nucleotide phosphodiesterase 1A‐like (*NPD*, ISCGN302940) and rap guanine nucleotide exchange factor 2‐like (*RAPGEF2*, ISCGN121770) were selected as target genes and for primer design because both displayed elevated copy numbers in females across two HiFi datasets: Female‐1 (~2000 kb) and Female‐2 (~1800 kb). These two candidate genes are highlighted in Table S1, where “Start” and “End” indicate the coordinates of variation, “Window” reflects the number of 1 kb segments, and shows copy number differences between sexes, summarized as male‐ or female‐enriched. Elongation factor 1‐alpha (*EF‐1α*) was identified as a reference gene because it has a similar copy number in both sexes and has been used as a normalization control in other tick species (Kim et al. 2023; Salata et al. 2020).

Primers and fluorescently labeled probes were designed using the *I. scapularis* genome (IscapMGN; GCA_031841145.1) as a reference (Table 1). Candidate gene sequences were selected and assessed for specificity using NCBI Primer‐BLAST, and additional off‐target screening was performed using NCBI BLAST against the *I. scapularis* genome. Amplicon lengths were kept between 70 and 150 bp to optimize qPCR performance. Probes were labeled with either 5′‐FAM or 5′‐HEX fluorophores and a 3′ ZEN Iowa Black quencher.

**TABLE 1:** Forward and reverse primer sequences for *I. scapularis* and *R. microplus* *NPD*, *EF‐1α*, and *RAPGEF2*, along with corresponding probe sequences for all three genes in *R. microplus* , used in PCR, qPCR, and ddPCR assays.

Primer	Sequence 5′‐3′
***I. scapularis***	
ISCGN‐*EF‐1α*‐F	CTGGAACGGTGACAACATGC
ISCGN‐*EF‐1α*‐R	GGGACTTGCCCTCAGACTTG
ISCGN‐*RAPGEF2*‐F	CGTTTGCCAACATGACCCTC
ISCGN‐*RAPGEF2*‐R	GTCGTTCATCACCACTGTGC
ISCGN‐*NPD*‐F	CAACGGTCACCAACAATGGC
ISCGN‐*NPD*‐R	TTTTCAGCTCCCGGTTGGAG
***R. microplus***	
*EF‐1α*‐F	CGTCTACAAGATTGGTGGCATT
*EF‐1α*‐R	CTCAGTGGTCAGGTTGGCAG
*RAPGEF2*‐F	CCCGTAGTCAAGAGGTCATGG
*RAPGEF2*‐R	CCTCCTCATCAGCCTCATGG
*NPD*‐F	GCAGAGGCGAGAAGAAAACG
*NPD*‐R	TGTAGGTTGCGGCTCAGTAG

Probe	Sequence 5′‐3′
***I. scapularis***	
ISCGN‐*EF‐1α*‐HEX	5′ HEX‐GGGCTGGTACAAGGGCTGG‐ZEN Iowa black 3′
ISCGN‐*NPD*‐FAM	5′ FAM‐GGGAAGAGCGGCAGTGC‐ZEN Iowa black 3′
***R. microplus***	
*EF‐1α*‐HEX	5′ HEX‐GAGACCGGTGTCCTCAAGC‐ZEN Iowa black 3′
*NPD*‐FAM	5′ 6FAM‐TGGAGTCAGGCTTGCTGAAG‐ZEN Iowa black 3′
*RAPGEF2*‐FAM	5′ 6FAM‐GCAACAGCAGCCTCTTATGC‐ZEN Iowa black 3′

Prior to performing any molecular assays, all primer sets were validated using conventional PCR and gel electrophoresis. Each reaction included extracted gDNA from a single leg of an individual adult male or female tick, Apex TaqRed master mix (Apex #42–137), the respective primer set, and nuclease‐free water. PCR cycling conditions are detailed in Table 2, and the PCR products were run on a 1.2% Tris‐acetate‐EDTA (TAE) gel.

**TABLE 2:** PCR, qPCR, and ddPCR cycling conditions, denaturing, annealing, and extension temperatures and time for PCR, qPCR, and ddPCR assays.

Step	Temperature	Time	Cycles
**PCR conditions**			
*I. scapularis*			
Initial denaturation	98°C	3 min	1
Denaturation	98°C	30 s	35
Annealing	61°C	50 s	
Extension	72°C	1 min	
Final extension	72°C	10 min	1
Hold	4°C	∞	
**qPCR conditions**			
Initial denaturation	95°C	10 min	1
Denaturation	95°C	11 s	40
Annealing	60°C	30 s	
Hold	12°C	∞	
**ddPCR conditions**			
Initial denaturation	98°C	3 min	1
Denaturation	98°C	30 s	40
Annealing	60°C	1 min	
Extension	98°C	10 min	
Hold	4°C	∞	
*R. microplus*			
Initial denaturation	95°C	3 min	1
Denaturation	95°C	10 s	40
Annealing	60°C	15 s	
Extension	72°C	15 s	
Melt curve	95°C	3 min	
	60°C	15 s	
	95°C	10 s	

#### 2.3.2 *Rhipicephalus microplus*

In *R. microplus* , CNV genes were identified on chromosomes 2 and 8. Similar to *I. scapularis* , the most consistently variable genes were identified as *NPD* (LOC119176070) and *RAPGEF2* (LOC119161094) from the reference genome (BIME_Rmic_1.3: GCF_013339725.1) (Table S2). Primers were designed using the primer design tool in Benchling [Benchling (Biology Software), 2025. Retrieved from https://benchling.com]. Candidate genes were then tested by SYBR Green qPCR to determine their copy number relative to a housekeeping gene known to be present in two copies in each sex—elongation factor 1‐alpha 1 (*EF‐1α*, LOC119170533). Each 10 μL qPCR reaction contained 5 μL qPCRBIO SyGreen Mix Lo‐ROX (PCR Biosystems, London, UK); 0.05 μM of each primer; 5 ng of template gDNA; and molecular‐biology‐grade water to make up the rest of the volume. Reactions were run on an AriaMX qPCR system (Agilent, Santa Clara, CA, USA) with thermocycler conditions as given in Table 2. Candidate genes were evaluated by calculating the relative copy number using a −ΔΔ*C*_t_ score (Livak and Schmittgen 2001) for 19 male and 13 female samples. An adult male sample was used as the calibrator (reference sample) as male naturally represents the one‐copy state, which is used as the baseline, using the following equation:

(1) $$\begin{array}{c} \text{Relative copy number}=\\ \frac{2^{\left(\text{candidate gene male calibrator}\;C_{\text{t}}-\text{candidate gene female sample}\;C_{\text{t}}\right)}}{2^{\left(\text{housekeeping gene male calibrator}\;C_{\text{t}}-\text{housekeeping gene female sample}\;C_{\text{t}}\right)}} \end{array}$$

where the calibrator is the reference male sample used for comparison, and the sample represents the test male or female individual. The best candidate genes were identified as those with scores closer to 2 in female samples and closer to 1 in male samples (Table S2). Probe‐based qPCR assays were designed using the probe designing tool in Benchling. Candidate gene probes were labeled with 5′‐FAM fluorophores, the housekeeping gene *EF‐1α* with a 5′HEX fluorophore, and both used 3′ ZEN Iowa Black quencher. Final primer and probe sequences for *NPD*, *RAPGEF2*, and *EF‐1α* are provided in Table 1.

### 2.4 Experimental Setup for Molecular Sexing Assays

Using three genes: *NPD*, *RAPGEF2*, and *EF‐1α*, identified with CNV in the *I. scapularis* genome, we used three molecular approaches: (1) SYBR Green‐based qPCR, (2) fluorescent probe‐based qPCR, and (3) ddPCR. Only fluorescent probe‐based qPCR was used as a sexing assay in *R. microplus* . We compared these approaches with the published duplex endpoint PCR (Ronai et al. 2025).

#### 2.4.1 Quantitative PCR (SYBR Green‐Based)

DNA from individual *I. scapularis* leg samples was used. Each SYBR‐based qPCR reaction was performed in a 10 μL volume containing 0.25 μM of the appropriate primer pairs (*NPD/RAPGEF2* and *EF‐1α*), 5 μL of SYBR Green Supermix (Bio‐Rad; Catalog #1725274), 3.7 μL of nuclease‐free water, and 0.5 μL of genomic DNA (~2.5 ng). All reactions were run in triplicate (CFX Connect Real‐Time System, BioRad), with each primer set assessed individually. PCR conditions are listed in Table 2. Although both *NPD* and *RAPGEF2* were initially tested, only *NPD* was used in subsequent *I. scapularis* nymph assays due to inconsistent results with *RAPGEF2* primers.

Relative copy number for each nymph was calculated using the $2^{-∆C_{t}}$ method (Schmittgen and Livak 2008), where Δ*C*_t_ = *C*_t_ (CNV gene) − *C*_t_ (housekeeping). CNV gene *C*_t_ values were normalized directly to the *EF‐1α* reference gene without using an adult calibrator. Samples with relative copy‐number values below 0.97 were classified as male, whereas values ≥ 0.97 were classified as female.

#### 2.4.2 Probe‐Based qPCR

For *I. scapularis* probe‐based qPCR reactions, the SYBR Green Supermix from the method above was replaced with 5 μL of PCR Biosystems PROBE Master Mix (Batch #240F225K09) and supplemented with 0.25 μM of each gene‐specific probe labeled with HEX and FAM fluorophores (Table 1).

For *R. microplus* , two duplex qPCR reactions were performed per sample on an AriaMX qPCR system (Agilent, Santa Clara, CA, USA). Each 10 μL reaction contained 5 μL of pPCR Biosystems Probe Mix Lo‐Rox (PCR Biosystems, London, UK); 0.05 μM of each forward and reverse primer (*NPD* or *RAPGEF2* and *EF‐1α*); 0.05 μM of each probe; 5 ng of template gDNA; and pure water to make up the rest of the volume. qPCR cycling conditions are outlined in Table 2. For probe‐based qPCR assays in both *I. scapularis* and *R. microplus* , the relative copy number was calculated using Equation (1) above.

#### 2.4.3 Digital Droplet PCR (ddPCR)

ddPCR was used only for the *NPD* gene, normalized to the housekeeping gene *EF‐1α*. Primer and probe sequences were designed based on gene models mapped to the *I. scapularis* Gulia‐Nuss genome (Nuss et al. 2023). Adult male and female gDNA samples were included as internal controls in each ddPCR assay to help with the classification of thresholds. ddPCR reactions were performed using the Bio‐Rad QX200 Droplet Digital PCR system and ddPCR Supermix for Probes (no dUTP) (Catalog #1863024). Different reaction formulations were assessed.

Each ddPCR reaction was prepared in a total volume of 22 μL, containing 11 μL of 2× ddPCR Supermix, 0.9 μM of each primer, 0.25 μM of each probe, and 2.2–4 μL of genomic DNA (0.5–0.91 ng/μL). The remaining volume was adjusted with nuclease‐free water. Reactions were mixed gently before proceeding to droplet generation.

For droplet generation, 20 μL of each prepared reaction mix was loaded into a DG8 cartridge, followed by the addition of 70 μL of droplet‐generation oil. After droplet formation using the Bio‐Rad AutoDG Droplet Generator, 40 μL of the generated droplet (sample) was transferred to a 96‐well PCR plate for amplification. PCR cycling conditions are outlined in Table 2.

Following amplification, droplets were read using the QX200 Droplet Reader. Data were analyzed with Bio‐Rad QX Manager software, and target‐to‐reference copy number ratios were calculated from the absolute quantification output. These ratios, along with the data from the other molecular methods, were used to classify the 23 nymphal samples as male, female, or unknown based on the relative enrichment of the *NPD* target.

#### 2.4.4 Duplex PCR

This method uses two primer pairs in a single reaction: one amplifies a 326‐bp male‐specific region, and the other amplifies a 406‐bp region of the autosomal *40S ribosomal protein S4‐like* gene. *I. scapularis* nymph samples showing both the 326‐bp and 406‐bp bands were identified as male, while those with only the 406‐bp band were identified as female, as described by (Ronai et al. 2025). The test samples consisted of nymphal leg DNA, and two adult males and one adult female were included as positive controls. A no‐template reaction was included as a negative control. Details on cycling conditions and additional methods are provided in (Ronai et al. 2025).

## 3 Results

### 3.1 Sex Determination Assays for *Ixodes scapularis* Nymphs

#### 3.1.1 Validation of Sex‐Specific Primers and DNA Extraction Efficiency Using Conventional PCR

For *I. scapularis*, the *RAPGEF2*, *NPD*, and *EF‐1α* genes were used for conventional PCR to test the amplification from gDNA extracted from the legs of male and female adults. All amplicons were detected at ~100–125 bp, as expected from the sequence, with minimal to no non‐specific bands (Figure S1). While some reactions showed lower expression, the overall results confirmed that the DNA extraction protocol using a single tick leg was effective and that the primers were gene‐specific (Figure S1).

*Nymphal DNA*: For all the following assays, DNA extracted from a single leg of an individual nymph (*n* = 23) was used. DNA quality was checked by the amplification of *β‐actin* gene, and all 23 nymphal samples were amplified successfully (Figure S2).

#### 3.1.2 Duplex PCR

The duplex PCR assay generated strong bands for nine of the 23 *I. scapularis* nymphal leg samples. Of these, seven displayed two bands corresponding to the male‐specific 326‐bp fragment and the 406‐bp autosomal fragment, indicating a male sex, while two leg samples showed only the 406‐bp autosomal band, indicating a female sex (Figure S3, Table 3). While some of the samples produced amplification, many bands were faint and difficult to interpret confidently, likely due to the limited gDNA available from a single nymph leg. Additionally, using higher gDNA amounts in the duplex PCR led to non‐specific bands, which made determining the sex even more challenging—an observation similar to (Koloski, Adam, et al. 2025; Koloski, Sharma, et al. 2025). This limitation prompted us to explore more sensitive and quantitative molecular approaches to improve accuracy and reliability in sex determination.

**TABLE 3:** Comparison of sex classification results across molecular assays.

Nymph sample	Duplex PCR	Probe‐based qPCR	SYBR‐based qPCR	ddPCR	Assigned sex	# of matching molecular methods
N1	M	—	M	—	M	2
N2	—	M	F	M	M	2
N3	M	M	M	M	M	4
N4	—	F	F	M	F	2
N5	—	F	F	F	F	3
N6	—	M	F	F	F	2
N7	—	F	F	F	F	3
N8	—	M	F	F	F	2
N9*	M	M	M	M	M	4
N10	—	—	F**	F	?	0
N11	M	M	M	M	M	4
N12	M	F	F	—	F	2
N13	—	M	F	F	F	2
N14	—	F	F	F	F	3
N15	—	M	F	F	F	2
N16	M	M	F	—	M	2
N17*	—	F	F	F	F	3
N18	F	M	M	—	M	2
N19	F	F	F	F	F	4
N20	M	F	M	—	M	2
N21*	—	F	F	F	F	3
N22	—	?	F	—	?	0
N23	—	F	F	F	F	3

*Note:* This table summarizes the concordance of sex classifications obtained from four molecular methods: duplex PCR, SYBR Green qPCR, probe‐based qPCR, and droplet digital PCR (ddPCR). Nymphal samples (N1–23) are grouped by putative sex based on molecular data, with “M” indicating male and “F” indicating female. Four nymphs showed full agreement across all four methods. Another six nymphs had matching results from three molecular methods, and 11 had matching results from two assays. Two nymphs (N10 and N22) were classified as undetermined (?). N10 produced a female result by ddPCR and an unusually high relative copy‐number value (7.23) by SYBR‐based qPCR that fell outside the validated female reference range and was therefore excluded from classification. N22 showed a female result by SYBR qPCR, whereas its probe‐qPCR value was close to the classification cutoff and was treated as indeterminate, with no additional molecular result available to resolve the discrepancy. Samples were sexed if results from at least two molecular methods agreed. Three nymphs, marked with an asterisk (*), died before initiating feeding. **Had a high value outside the cutoff so predicted female but was considered an outlier.

#### 3.1.3 SYBR‐Based qPCR

To evaluate the SYBR Green qPCR approach, we initially tested 13 adult males and 13 adult females to establish a baseline amplification and confirm the reliability of the method before applying it to immature life stages (Figure 1A,B). This step was essential to ensure the accuracy of our results when testing nymph samples. This method was then applied to 23 nymphal samples for validation.

**FIGURE 1:**
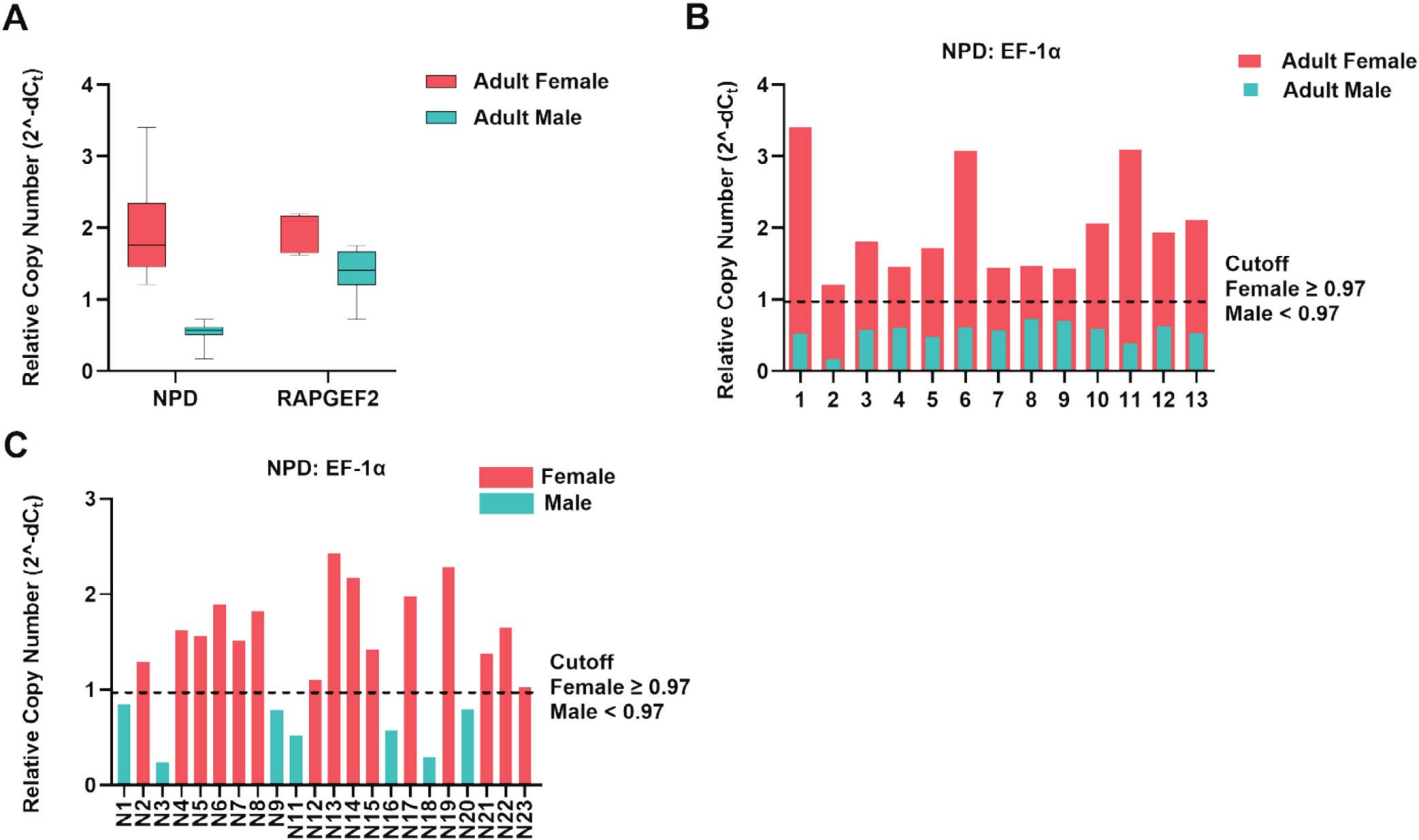
Comparison of fold change variation between male and female genomic DNA samples using SYBR Green based qPCR assay. (A) Boxplot of relative copy number ratios ($2^{-∆C_{t}}$) of the NPD (*n* = 13) and RAPGEF2 (*n* = 11) genes in adult male and female *I. scapularis* . EF‐1α was used as the reference gene. RAPGEF2 exhibited partial overlap between male and female samples, whereas NPD showed a clear separation between the sexes. (B) relative copy number ratios ($2^{-∆C_{t}}$) of the *NPD* gene normalized to *EF‐1α* in individual adult *I. scapularis* . Based on the distribution of the adult controls, a cutoff value of 0.97 was established, with samples having relative copy number ratios < 0.97 classified as males and ≥ 0.97 classified as females. (C) DNA from a single leg collected individually from 23 *I. scapularis* nymphs was used for qPCR. Relative Copy number ratio was calculated using the $2^{-∆C_{t}}$ method. *NPD* was used as the target gene and *EF‐1α* as the reference gene.

The assay for adult *I. scapularis* samples yielded a ratio of the sex‐specific gene of interest to the housekeeping *EF‐1α* gene for males that ranged from 0.72 to 1.76 when using *RAPGEF2* as the gene of interest and from 0.16 to 0.72 when using *NPD relative to EF‐1α* (Table S3). Using RAPGEF2, male and female samples showed partial overlap in their relative copy number ratios, although all male values remained lower than the average female control. In contrast, NPD showed a clearer separation between male and female samples (Figure 1A). These data matched the CNV identified in the *I. scapularis* genome, which showed both genes of interest being enriched in the females (Table S1). Following validation with adult *I. scapularis* samples, both SYBR and probe‐based qPCR methods were applied to nymphs. Adult male and female gDNA were included in both methods as positive controls to determine thresholds and to establish baseline amplification patterns for probe‐based qPCR.

Of the two target genes initially tested on *I. scapularis* nymphs, *RAPGEF2* showed overlapping relative copy number distributions between males and females and was therefore excluded from further analysis; and only *NPD* was used. The qPCR results for the nymphs showed CNV patterns similar to those previously observed in adults. Based on the adult *NPD* distributions, a relative copy number cutoff of 0.97 was established, corresponding to the midpoint between the maximum male (0.72) and minimum female (1.21) values (Figure 1B, Table S3). Samples with relative copy number values < 0.97 were classified as male, whereas values ≥ 0.97 were classified as female (Figure 1C). Using this approach alone, we could confidently predict the sex of 22 out of 23 nymphs (95.6%) (Figure 1C, Table 3). One sample, N10 produced a female classification; however, its relative copy number (7.23) was outside the validated female reference range.

#### 3.1.4 Probe‐Based qPCR

For probe‐based qPCR in *I. scapularis* , the copy number was assessed using the *NPD*: *EF‐1α* copy number ratio. Samples with ratios < 1.66 were interpreted as male, while ratios ≥ 1.66 were considered female. These threshold values were based on the distribution of *NPD*: *EF‐1α* ratios obtained from adult male and female ticks of known sex (Figure 2A). The cutoff value of 1.66 was determined as the midpoint between the highest adult male (1.59) and the lowest adult female (1.75) relative copy number values obtained from positive control samples of known sex (Figure 2A, Table S3). This method allowed us to classify 20 out of 23 samples (87%), and 13 of which matched the classifications obtained from SYBR Green‐based qPCR data (Figure 2B, Table 3). These results further support *NPD* as a reliable target for molecular sexing of *I. scapularis* nymphs.

**FIGURE 2:**
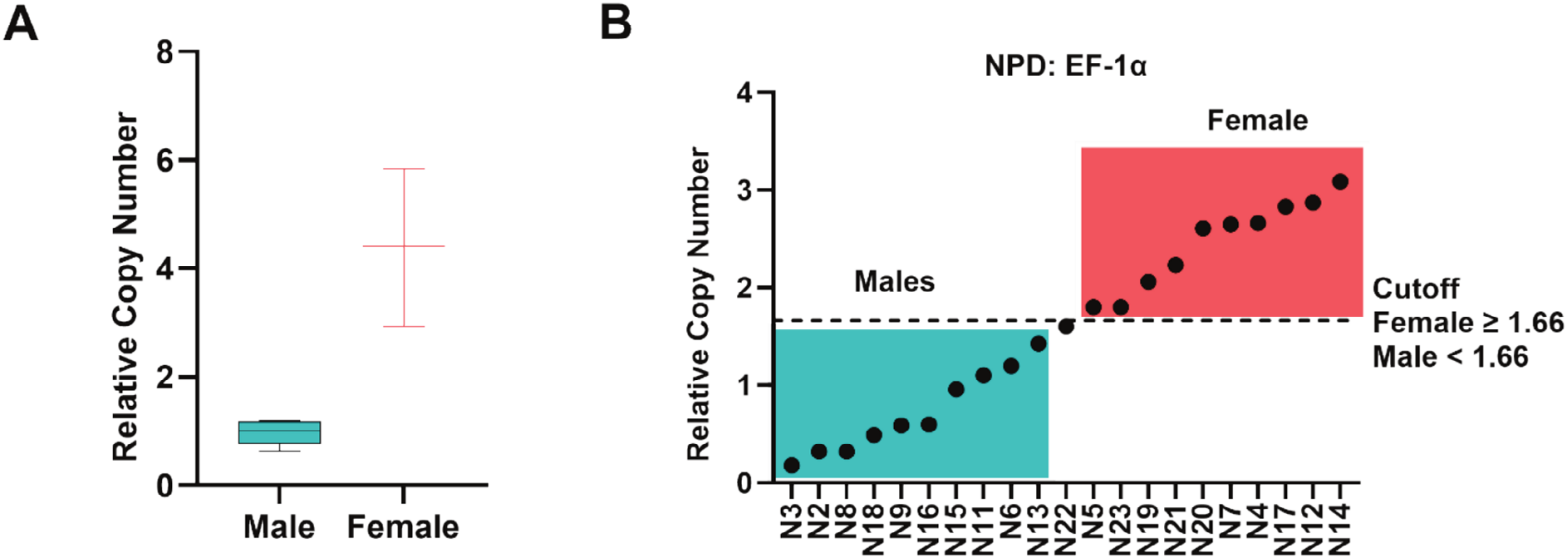
Probe‐based qPCR assay to determine sex of *I. scapularis* nymphs. (A) Boxplot of relative copy number ratios of the *NPD* gene in adult males (*n* = 5) and females (*n* = 3) *I. scapularis* determined by probe‐based qPCR using EF‐1α as the reference gene. (B) DNA from a single leg collected individually from 23 nymphs was used for qPCR. Each point represents an individual nymph sample, with copy number values calculated relative to the reference gene *EF‐1α*. Thresholds were established using adult male and female samples to interpret copy number results: Samples with an *NPD*: *EF‐1α* ratio < 1.66 were classified as male, whereas those with ratios ≥ 1.66 were classified as female. Using this approach, 20 samples were confidently classified, 10 as male, 10 as female, and 1 sample was difficult to assign because of the value close to the cutoff. Two samples (N1 and N10) produced unusually high copy number values (above 5) and were excluded from the analysis as outliers. N22 produced a value close to the cutoff and was treated as indeterminate.

#### 3.1.5 ddPCR

To further validate the sex of the *I. scapularis* nymphal samples, ddPCR was used to measure *NPD* ratio levels, normalized to the *EF‐1α* reference gene. Adult male and female gDNA were included in each run as internal controls to help set classification thresholds. Different reaction conditions were evaluated, and variability between replicates was reduced by increasing the gDNA input. Initial reactions (22 μL) used 1 μL of genomic DNA (0.23 ng/μL) with standard primer (0.9 μM) and probe (0.25 μM) concentrations but showed high variability and large error bars (Figure S4). To improve precision, we optimized the assay by increasing the genomic DNA input to 2.2 μL and 4 μL (0.51–0.92 ng/μL) and adjusted primer and probe concentrations accordingly. The 2.2 μL input followed Bio‐Rad's recommended protocol. All results were generated using these optimized conditions.

A copy number ratio of *NPD* to *EF‐1α* of < 0.6 was classified as male, while ratios ≥ 1.0 were considered female, based on consistent patterns observed in adult controls (Figure 3A, Table S3). Using this method, we were able to confidently classify 17 of the 23 (74%) nymphal samples as male or female (Figure 3B). Of these, 15 samples matched classifications from at least one of the SYBR or probe‐based qPCR methods, supporting the reliability of molecular tools for sex determination in *I. scapularis* nymphs (Table 3).

**FIGURE 3:**
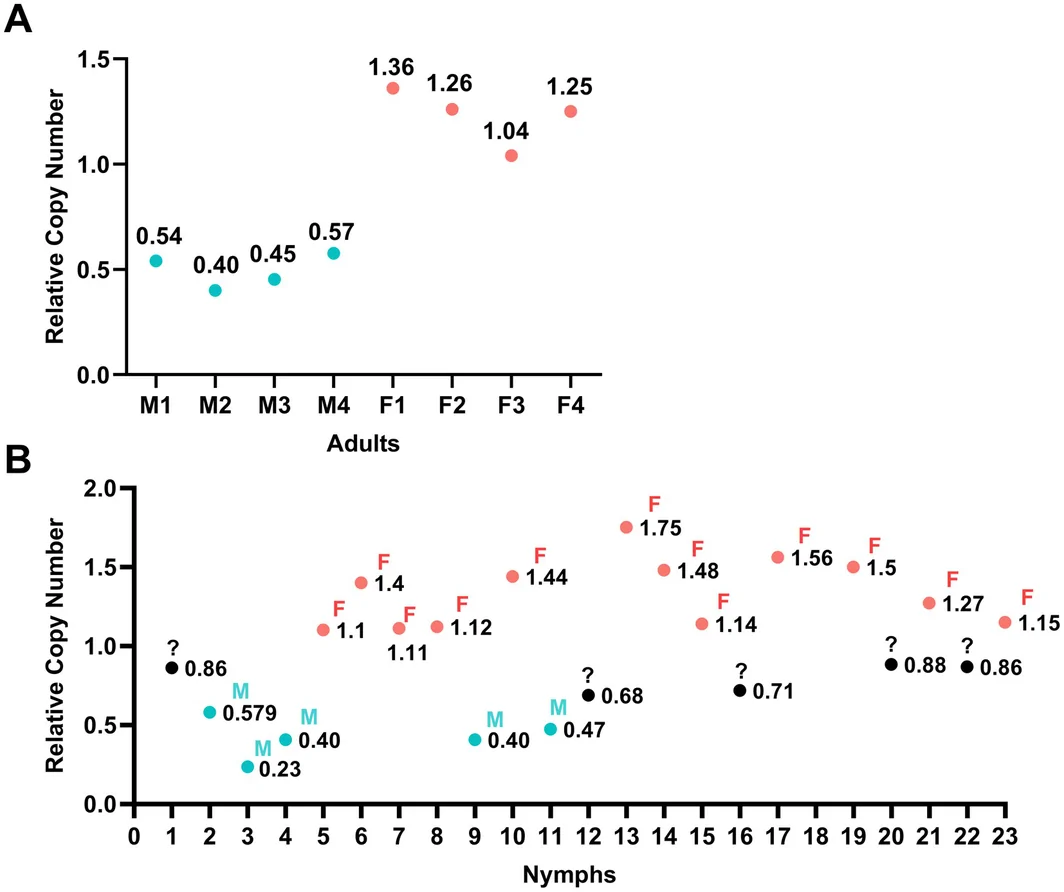
ddPCR assay to determine sex *of Ixodes scapularis*. (A) Adult control samples (four males and four females) were used to establish thresholds. Males consistently showed *NPD*: *EF‐1α* ratios < 0.6, while females showed ratios ≥ 1.0. (B) Nymphal samples were analyzed using 2.2–4 μL (0.5–0.91 ng/μL) of gDNA per reaction. Based on the thresholds established from adult controls (males; < 0.6 and females: ≥ 1.0), 17 of the 23 samples were confidently classified as male or female (five classified as male and 12 as females). Five samples had intermediate ratios (0.6– < 1.0), and one sample (N18) had no ddPCR result.

#### 3.1.6 Comparison of Molecular Sexing Methods and Morphological Validation of *I. scapularis* Nymphs

To assess the accuracy and consistency of our molecular assays, we compared results from all four molecular methods (duplex PCR, SYBR Green qPCR, probe‐based qPCR, and ddPCR). Samples were grouped by putative sex: male (M), female (F), or undetermined (?), based on the molecular data (Table 3). Sex was assigned when at least two molecular methods produced consistent results. For example, if both SYBR and ddPCR identified a sample as female while the probe‐based method failed or gave conflicting results, the sample was classified as female. This consensus‐based approach helped reduce the impact of technical variability from any single method.

Of the 23 nymphal legs tested, we were able to assign sex to 21 individuals (91.3%)—eight males and 13 females—based on consistent results from at least two molecular methods. Four nymphs showed complete agreement across all four assays. An additional six nymphs had matching results from three methods, and 11 more showed agreement between two. The remaining two samples (N10 and N22) could not be confidently classified (Table 3). N10 could be classified as female because it produced a female result by ddPCR but yielded an unusually high SYBR‐based relative copy‐number value that fell outside the validated adult female reference range, whereas N22 was classified as female by SYBR qPCR, whereas its probe‐qPCR value was close to the cutoff and was therefore treated as indeterminate (Table 3).

#### 3.1.7 Morphological Validation of Sex

Of the initial 23 nymphs, 20 survived until ready to feed (~6 weeks), while three died prior to feeding. Of these three, one was classified as male and two as females, according to results from four assays (Table 3). Of the 20 surviving nymphs, 10 fed to repletion and seven molted successfully, allowing for morphological verification of sex and evaluation of assay accuracy (Figure 4A).

**FIGURE 4:**
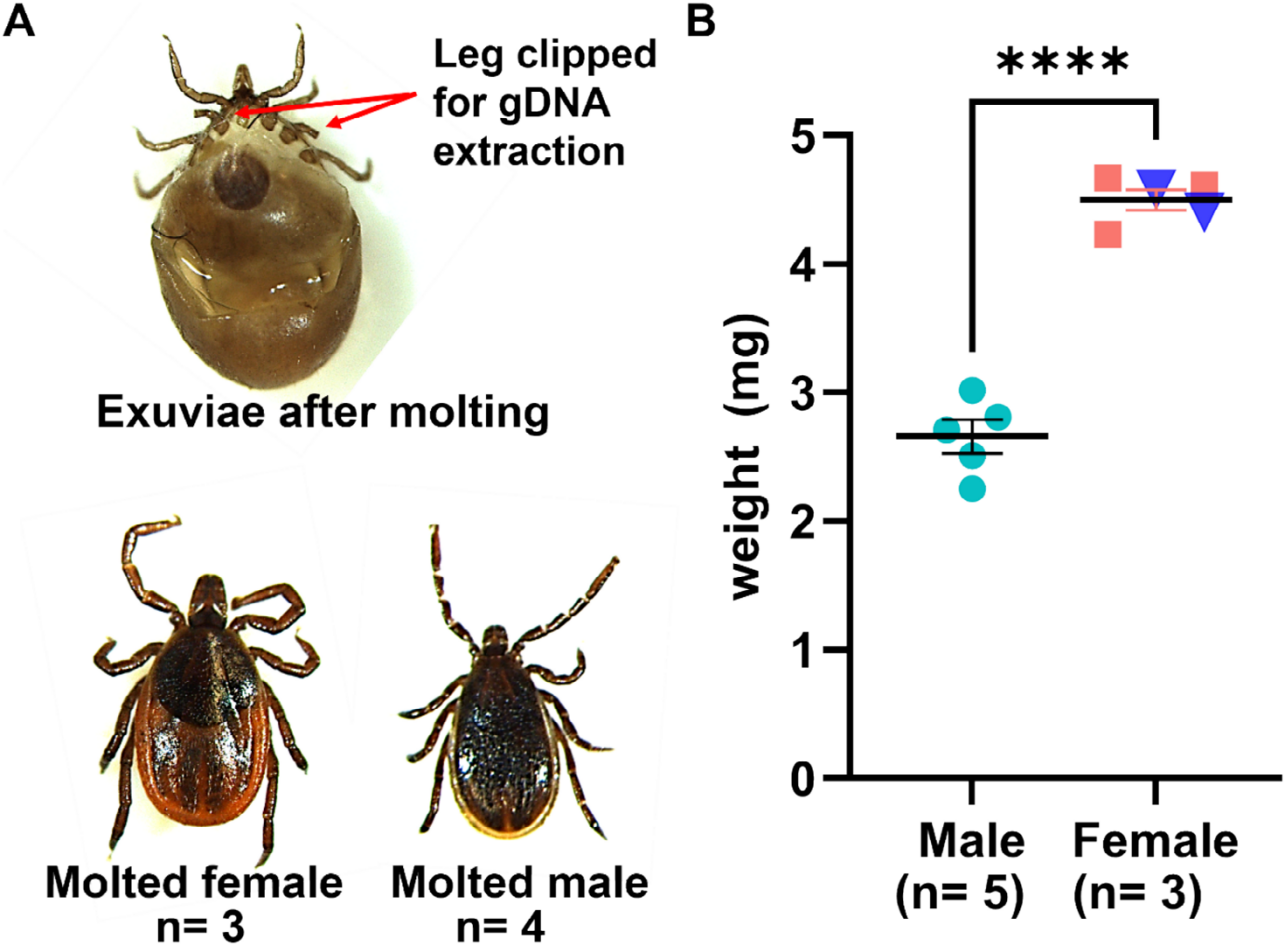
Adult *Ixodes scapularis* molted from the molecularly sexed nymphs. (A) Exuviae (molted casing) left behind after an *I. scapularis* nymph molted to the adult stage. The arrow indicates the location where a single leg was clipped for non‐lethal DNA extraction; the missing leg regenerated in the resulting adult. The representative image shows 2nd and 7th legs clipped, to establish that leg clipping does not impact survival and blood feeding. Representative image of adult male (right) and adult female (left) that successfully molted from nymphs whose sex was determined using the CNV‐based molecular assays. (B) Weight of blood‐fed *I. scapularis* nymphs. Ten blood‐fed nymphs were weighed, including five assigned as males, three assigned as females, and two undetermined by the molecular assay. Males are represented by circles, females by squares, and the undetermined nymphs by blue inverted triangles. Both undetermined nymphs fell within the female body weight range and were therefore provisionally classified as females based on body weight. Data are presented as mean ± SEM Statistical significance was calculated by unpaired *t*‐test with Welch's correction was used for comparing control using GraphPad Prism v10. ****p < 0.0001.

For nymphs predicted to be male by molecular methods, 4 of 5 (80%) that survived to molting emerged as adult males. One assigned male nymph died 2 months post‐blood‐meal, before molting. For those predicted to be female, 2 of 3 (66.6%) individuals molted into an adult female; one of them died after blood meal. Despite the small number of individuals that molted, these results provide preliminary morphological support for the molecular sex classification.

#### 3.1.8 Nymphal Body Weight as a Predictor of Sex

We also tested whether body weight could be used to predict sex. The body weights of the 10 blood‐fed nymphs that survived feeding were measured, including five molecularly assigned males and three molecularly assigned females (Figure 4B). The body weights of the molecularly assigned males and females formed two distinct groups, with females weighing significantly more than males (*p* < 0.0001) (Figure 4B). Nymphs that could not be sexed molecularly (undetermined, *n* = 2) were assigned to male or female categories based on body weight. These two unclassified nymphs fell within these ranges, allowing us to designate these two as likely female. Of these, one weight‐assigned female died after feeding (~2 months), but the other survived to molt and confirmed the predicted sex, supporting the utility of weight as an additional indicator.

### 3.2 Sex Determination Assay for *Rhipicephalus microplus* Nymphs

#### 3.2.1 Probe‐Based qPCR

In *R. microplus* , two dual probe‐based assays were similarly designed to target *RAPGEF2* and *NPD* respectively, with *EF‐1α* as the reference gene. These assays were validated and optimized using sexed adult male and female ticks. The mean relative copy number ratio of *NPD* was 0.99 in males and 2.02 in females, whereas that for *RAPGEF2* was 0.81 in males and 1.41 in females (Figure 5A). While both the *NPD* and *RAPGEF2* assays individually produced reliable results, using them together helped boost confidence in the findings.

**FIGURE 5:**
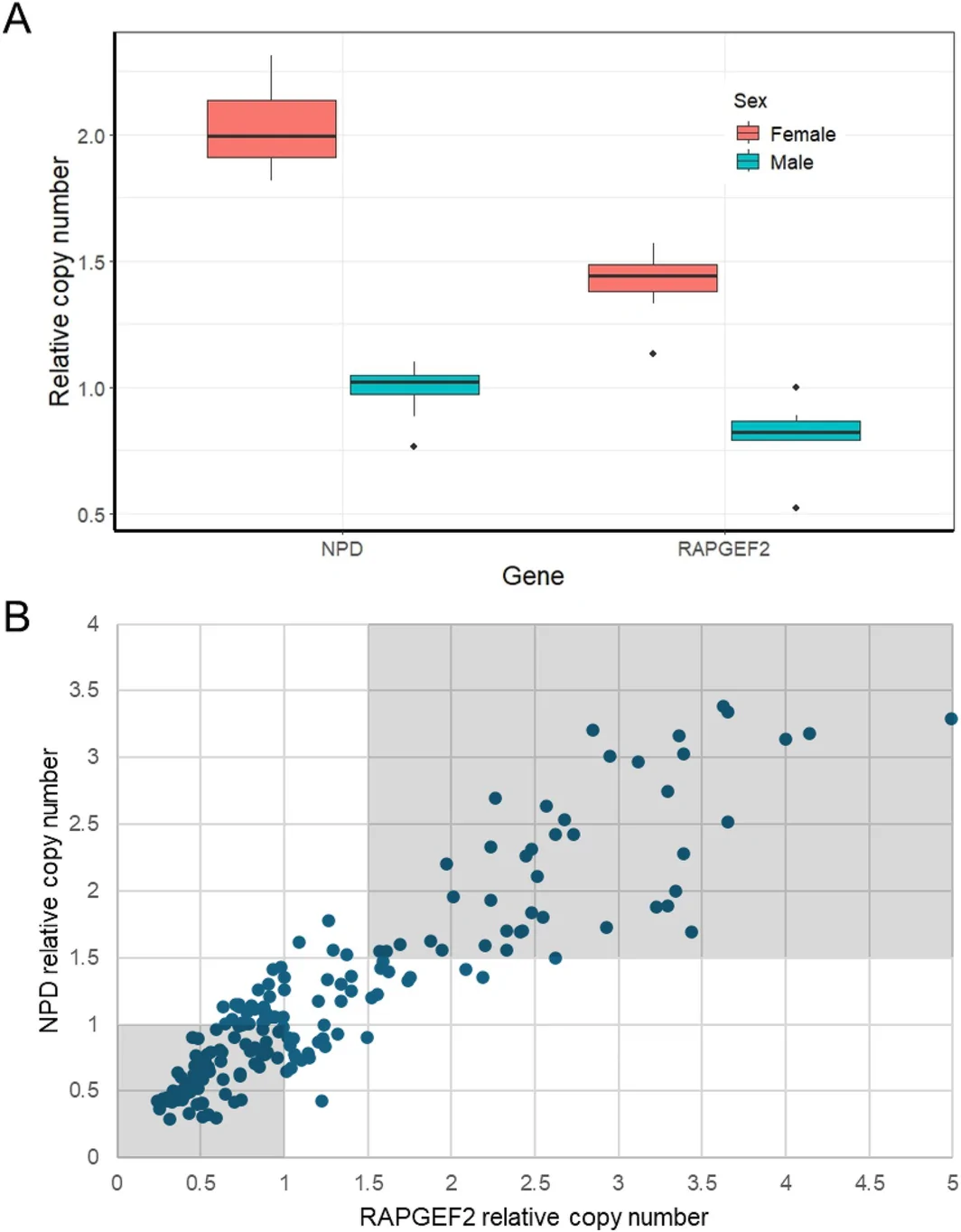
Probe based qPCR assay to determine sex *of R. microplus*. (A) Boxplot of relative copy number of genes *NPD* and *RAPGEF2* in male and female adult *R. microplus* . Values were obtained from a dual probe qPCR assay using *EF‐1α* as a housekeeping gene. (B) Results of dual probe‐based qPCR assay on unsexed *R. microplus* nymphs. Both *NPD* and *RAPGEF2* gene assays were used to sex 201 nymphs. Each point represents an individual nymph sample, with copy number values of *NPD* or *RAPGEF2* calculated relative to *EF‐1α*. Thresholds were established using adult male and female samples to interpret copy number results. Samples with relative *NPD* and *RAPGEF2* copy number under 1 were classified as male and above 1.5 were classified as female. Values between these thresholds were considered undetermined.

When using this assay to sex nymphs, we used a more conservative window of acceptable values, as samples returned a wider range of copy number values than adult controls. Any sample that produced a *C*_t_ value of 32 or above was omitted from the assay as it was assumed to have low template quality. Samples with a relative copy number of above 0.5 and below 0.2 were also omitted from sexing. Samples with both relative *NPD* or *RAPGEF2* copy numbers below 1 were considered to be male while those above 1.5 were considered to be female. Sex determination of individuals displaying intermediate copy number values was determined unreliable (Figure 5B). Out of 201 nymphs tested with these assays, six samples were discounted due to high *C*_t_ values. Nine samples were discounted due to too high or too low relative copy numbers. Of the remaining samples, 78 nymphs were assessed as male, and 42 were as female. The remaining 66 nymphs had intermediate copy numbers which could not be unequivocally assigned to either sex. Copy number results from the *NPD* assay fell into a more bimodal distribution than that of *RAPGEF2* (Figure S5), and there was a larger gap in mean copy number among adult samples in the *NPD* assay. Therefore, the single *NPD* assay may be reliable enough to be used on its own.

## 4 Discussion

This study highlights the development of molecular sex identification methods for *I. scapularis* and *R. microplus* nymphs using a female‐enriched gene, calcium/calmodulin‐dependent 3′,5′‐cyclic nucleotide phosphodiesterase 1A‐like (*NPD*), detected via SYBR Green qPCR, probe‐based qPCR, and droplet digital PCR (ddPCR). As shown for *I. scapularis* , these approaches require only a small amount of tissue, such as a single leg or a portion of a leg, making it possible to determine sex without sacrificing the tick, which allows for continued monitoring or downstream validation. To our knowledge, this is the first adaptation of high‐sensitivity molecular sexing assays, such as qPCR and ddPCR for immature stages in any tick species, addressing a critical gap since sex can typically not be distinguished morphologically until after molting to adults.

Accurate and early sex identification of ticks is important for understanding population sex ratios. Early sex identification also enables researchers to study how male and female ticks differ in traits like host‐seeking, blood‐feeding, and pathogen acquisitionfactors that directly influence their role in disease ecology (Kiszewski et al. 2001; Koloski, Adam, et al. 2025; Koloski, Sharma, et al. 2025; Lefcort and Durden 1996; Troughton and Levin 2007; Varone et al. 2025). Additionally, early sexing is essential for applying genetic control strategies involving self‐sexing strains, which have been successful in mosquitoes but remain underdeveloped in ticks (Feng et al. 2021; Kyrou et al. 2018; Spinner et al. 2022; Wang et al. 2021).

Previous efforts to sex immature ticks often rely on destructive dissection, high‐end microscopy, or genetic markers not yet validated in qPCR‐based platforms (Hu and Rowley 2000; Keirans et al. 1996). Recently, Ronai et al. (2025) developed a duplex PCR‐based method for molecular sexing in *I. scapularis* , that targets both a male‐specific and an autosomal fragment, enabling sex determination in immature stages. However, when we applied this method using non‐lethal DNA extraction from a single leg, the resulting bands were faint and inconclusive (Figure S3). Attempts to optimize the protocol by increasing either the amount of gDNA or the number of PCR cycles led to the appearance of spurious bands, ultimately making the method unreliable and inconsistent for our non‐lethal sampling approach (our data and Koloski, Adam, et al. 2025; Koloski, Sharma, et al. 2025). This outcome prompted us to pursue the development of more sensitive techniques for molecular sex determination.

By targeting a gene known to have a higher copy number in females (*NPD*), we successfully classified unfed nymphs according to their sex using sensitive qPCR methods, including SYBR Green and fluorescent probe‐based assays (Figures 1, 2). This approach builds on the growing genomic resources available for *I. scapularis* (De et al. 2023; Nuss et al. 2023) and expands their use *to R. microplus* as well, supporting cross‐species application of the molecular sexing techniques outlined here. Compared to conventional PCR, qPCR offers several advantages because it is more sensitive, cost‐effective, and high throughput, making it a practical choice for routine sex determination. A similar qPCR‐based method has been successfully used to determine sex in a variety of organisms, including insects such as lepidopterans (Belousova et al. 2019), soft‐shell turtles (Literman et al. 2014), and pigs and lizards (Ballester et al. 2013; Rovatsos and Kratochvíl 2017). These studies demonstrate that qPCR is a reliable and widely applicable tool for sex identification across different species.

Among the molecular methods evaluated, ddPCR provided the most precise and consistent quantification of *NPD* copy number, largely due to its partitioning of template DNA into thousands of droplets, which enables absolute quantification without relying on standard curves. In comparison, qPCR methods were effective but displayed higher variability, most noticeably when template concentration was low. SYBR‐based qPCR outperformed probe‐based qPCR assays in terms of categorizing nymphal samples molecularly, but both were less sensitive than ddPCR. These findings align with previous studies showing that ddPCR often offers better reproducibility and a stronger tolerance to PCR inhibitors in biological samples (Sedláková et al. 2022). Using two genes instead of one, as we showed for *R. microplus* , led to higher confidence in the qPCR assay, suggesting that combining another gene with *NPD* from our CNV list may result in a more sensitive qPCR assay.

Additionally, we measured the body weight of engorged *I. scapularis* nymphs to determine whether there are sex‐based differences in weight post‐engorgement, as reported in previous studies (Hu and Rowley 2000). A number of studies have demonstrated that in *I. scapularis* and *I. ricinus* , engorged female nymphs weigh more than engorged male nymphs (Dusbabek 1996; Kahl et al. 1990; Koch 1981; Koloski, Adam, et al. 2025; Koloski, Sharma, et al. 2025). We attempted to classify the two samples that could not be sexed using molecular methods. According to their body weights, these two samples were classified as females; however, we could only validate one female as the other female nymph died before adult eclosion.

A limitation of this study is that validation of the *I. scapularis* assay was based on a relatively small number of nymphs that successfully molted into adults, which limited our ability to accurately estimate classification performance. Likewise, approximately one‐third of *R. microplus* nymphs remained unclassified because they did not meet the predefined confidence thresholds. These conservative thresholds were applied to minimize incorrect sex assignments, resulting in a trade‐off between classification rate and confidence.

Our work here establishes a foundation for developing a pan‐tick sex determination assay. Given that this assay works for both XX:XY and XX:XO systems, it will be interesting to explore whether these markers can be used across additional species. Interestingly, in *R. microplus* , several genes on chromosomes previously annotated as chromosomes 2 and 8 appeared to have a variable copy number. This result aligns with a recent karyotyping and genome reassembly of *R. microplus* by (Tidwell et al. 2024), in which previously annotated chromosomes 2 and 8 were identified as the X chromosome.

Future studies should explore larger datasets to test the robustness of these assays. Additional genes may also be identified to develop multiplex assays that improve accuracy and throughput. A deeper examination of the *I. scapularis* sex chromosomes, particularly the male‐specific and shared (pseudoautosomal) regions, could aid in identifying new genetic markers.

## 5 Conclusion

The non‐lethal molecular method described in this study enables sex determination from a single leg using a female‐enriched gene target, NPD, via SYBR Green qPCR, probe‐based qPCR, and ddPCR. This approach is minimally invasive, scalable, and preserves the viability of the tick for downstream analyses, such as behavioral assays, pathogen acquisition/transmission studies, or colony breeding. Importantly, this method applies to both *I. scapularis* and *R. microplus* , demonstrating its versatility across distantly related tick species. By combining sensitivity, species applicability, and non‐lethal sampling, our method provides a valuable tool for developmental, ecological, and vector biology studies where sex identification at early life stages is essential.

## Author Contributions

**Arvind Sharma:** formal analysis (equal), investigation (equal), methodology (lead), supervision (supporting), validation (lead), writing – original draft (equal), writing – review and editing (supporting). **Benjamin Faustino:** formal analysis (supporting), investigation (equal), methodology (equal), validation (supporting), writing – original draft (supporting). **Isaac Hinne:** data curation (supporting), formal analysis (supporting), methodology (supporting), resources (equal). **Jakub Wudarski:** data curation (supporting), formal analysis (supporting), methodology (supporting), resources (supporting), supervision (supporting). **Saransh Beniwal:** methodology (supporting), resources (supporting), validation (supporting). **Nurul Hamidi:** conceptualization (supporting), data curation (supporting), formal analysis (supporting), methodology (supporting), validation (supporting), visualization (supporting). **Kyla Ercit:** data curation (supporting), formal analysis (supporting), investigation (supporting), methodology (supporting), validation (supporting), visualization (supporting). **Sanjay Basu:** conceptualization (supporting), formal analysis (supporting), investigation (supporting), methodology (supporting), resources (supporting), supervision (equal), validation (supporting), visualization (supporting). **Malimba Lisulo:** conceptualization (supporting), formal analysis (equal), investigation (supporting), methodology (supporting), supervision (supporting). **Mattia Poletto:** conceptualization (equal), investigation (equal), methodology (equal), resources (equal), supervision (equal), validation (equal), visualization (equal), writing – original draft (equal), writing – review and editing (equal). **Kelly J. Matzen:** conceptualization (equal), data curation (equal), formal analysis (equal), funding acquisition (equal), investigation (equal), methodology (equal), project administration (equal), supervision (equal), writing – original draft (equal), writing – review and editing (equal). **Monika Gulia‐Nuss:** conceptualization (lead), data curation (equal), formal analysis (equal), funding acquisition (lead), investigation (equal), methodology (equal), project administration (lead), resources (equal), supervision (equal), validation (equal), visualization (equal), writing – original draft (equal), writing – review and editing (equal).

## Funding

This work was supported by the Bill and Melinda Gates Foundation (INV‐025721 and INV‐045130); the National Institutes of Health (R01AI172943, R21AI128393, R21AI139778, R21AI176352); and the Directorate for Biological Sciences (2019609).

## Conflicts of Interest

The authors declare no conflicts of interest.

## Supporting information


**Figure S1:** Visualization of amplified PCR products on a 1.2% agarose gel. PCR amplification of *EF‐1α*, *NPD*, and *RAPGEF2* in extracted adult male (M) and female (F) leg gDNA. The housekeeping gene actin was included as a positive control. The expected product size is ~90–130 bp for all primers, with actin expected to be slightly smaller (~90 bp) than the *EF‐1α*, *NPD*, and *RAPGEF2*. For size reference, an Econ low DNA ladder (100 bp–1000 bp) was used.
**Figure S2:** PCR amplification of the actin housekeeping gene in *I*. *scapularis* nymph DNA samples (N1‐23). The two unclassified samples (N10, and N22) produced clear actin amplicons, indicating good DNA quality. For size reference, DNA ladder II (100 bp–1000 bp) was used.
**Figure S3:** Duplex PCR assay for *I*. *scapularis* nymphs. Twenty‐three nymphs (lanes 1–23) using a published protocol (Ronai et al. 2025) targeting a male‐specific genomic region (326 bp) and an autosomal gene (406 bp) was used. Two adult males (M) and one adult female (F) were included as positive controls. Nymphs displaying both the 326‐bp and 406‐bp bands were classified as male (red letter M), while those showing only the 406‐bp band were classified as female. A 100–1000 bp DNA ladder II was used for size reference.
**Figure S4:** ddPCR results showing *NPD*: *EF‐1α* copy number ratios for *I. scapularis* nymphal samples with high variability. Nymphal samples (*n* = 23) were analyzed using 1 μL of gDNA per reaction. This setup produced high variability, and many of the samples displayed large error bars.
**Figure S5:** Histograms of relative copy numbers of genes *RAPGEF2* and *NPD* in unsexed *R.* *microplus* nymphs. A total of 201 unsexed nymphs were tested with a dual probe copy number qPCR assay to determine sex via copy number variation. Both assays produced a roughly bimodal distribution, indicating that these genes likely vary in copy number between males and females.
**Table S1:** Candidate genes exhibiting sex‐specific copy number variation in the *I. scapularis* Gulia‐Nuss genome. The table shows the chromosome, gene ID, fold change, and sex‐specific enrichment based on comparisons between male read depth and two female HiFi genomes (Female‐1: ~1000 kb, Female‐2: ~1800 kb). (*) indicate the genes used in this study.
**Table S2:** Chromosome, gene ID, and location based on genome assembly BIME_Rmic_1.3: GCF_013339725.1, and the mean fold change of copy number of the genes in adult females relative to male *R. microplus* . Several genes on chromosomes 2 and 8 show a higher copy number in females than in males. (*) indicate the genes used to sex nymphs in this study (*RAPGEF2* and *NPD*). (**) indicate *EF‐1α* which was used as a housekeeping gene.
**Table S3:** Descriptive statistics and classification thresholds for the SYBR‐based and probe‐based qPCR assays used to determine sex in adult *I. scapularis* . Relative copy number ratios were calculated using $2^{-∆C_{t}}$ with EF‐1α as the reference gene. Classification cutoffs were derived from the distribution of adult male and female control samples.

## Data Availability

All data supporting this study are included in the manuscript and Supporting Information.
