# Supplementary material for: Copy Number Variation‐Based Molecular Sexing of Ixodes scapularis and Rhipicephalus microplus Immature Stages Using qPCR and ddPCR Approaches
Source: Ecol Evol. 2026 Oct 28;16(11):e74562. doi: 10.1002/ece3.74562 (PMC13715080; doi:10.1002/ece3.74562)
Supplement: Supplementary file 1 [file ECE3-16-e74562-s001.docx]

**Copy Number Variation-Based Molecular Sexing of *Ixodes scapularis* and *Rhipicephalus microplus* Immature Stages Using qPCR and ddPCR Approaches**

Arvind Sharma^1¶^, Benjamin Faustino^1¶^, Isaac Hinne^1^, Jakub Wudarski^1^, Saransh Beniwal^1^, Nurul Hamidi^2^, Kyla Ercit^2^, Sanjay Basu^2^, Malimba Lisulo^3^, Mattia Poletto^2^, Kelly J. Matzen^2*^, Monika Gulia-Nuss^1^*

^1^Department of Biochemistry, Molecular Biology, and Biotechnology, University of Nevada, Reno, Reno, NV, USA

^2^Oxitec Ltd, 71 Innovation Drive, Milton Park, Abingdon, OX14 4RQ, UK

^3^Division of Immunology, The Roslin Institute, University of Edinburgh, Easter Bush Campus, Midlothian, EH25 9RG, United Kingdom

^¶^Equal contribution

*Corresponding authors

[mgulianuss@unr.edu](mailto:mgulianuss@unr.edu)

[Kelly.Matzen@oxitec.com](mailto:Kelly.Matzen@oxitec.com)

**Fig S1:**

**Fig S1: Visualization of amplified PCR product on a 1.2% Agarose Gel.** PCR amplification of *EF-1α*, *NPD*, and *RAPGEF2* in extracted adult male (M) and female (F) leg gDNA. The housekeeping gene actin was included as a positive control. The expected product size is ~90-130 bp for all primers, with actin expected to be slightly smaller (~90 bp) than the *EF-1α*, *NPD*, and *RAPGEF2*. For size reference Econ low DNA ladder (100 bp – 1000 bp) was used.

**Fig S2:**

**Fig S2: PCR amplification of the actin housekeeping gene in *I. scapularis* nymph DNA samples (N1-23).** The two unclassified samples (N10, and N22) produced clear actin amplicons, indicating good DNA quality. For size reference DNA ladder II (100 bp – 1000 bp) was used.

**1000 bp**

**1000 bp**

**Fig S3:**

**Fig S3: Duplex PCR assay for *I. scapularis* nymphs**. Twenty-three nymphs (lanes 1–23) using a published protocol (Ronai et al., 2025) targeting a male-specific genomic region (326 bp) and an autosomal gene (406 bp) was used. Two adult males (M) and one adult female (F) were included as positive controls. Nymphs displaying both the 326-bp and 406-bp bands were classified as male (red letter M), while those showing only the 406-bp band were classified as female. A 100-1000 bp DNA ladder II was used for size reference.

**Fig S4:**

**Fig S4: ddPCR results showing *NPD*: *EF-1α* copy number ratios for *I. scapularis* nymphal samples with high variability**. Nymphal samples (n= 23) were analyzed using 1 µL of gDNA per reaction. This setup produced high variability and many of the samples displayed large error bars.

**Fig S5:**

**Fig S5: Histograms of relative copy numbers of genes *RAPGEF2* and *NPD* in unsexed *R. microplus* nymphs.** A total of 201 unsexed nymphs were tested with a dual probe copy number qPCR assay to determine sex via copy number variation. Both assays produced a roughly bimodal distribution, indicating that these genes likely vary in copy number between males and females.

**Tables**

**Table S1**

**Table S1. Candidate genes exhibiting sex-specific copy number variation in *I. scapularis* Gulia-Nuss genome.** Table shows chromosome, gene ID, fold change, and sex-specific enrichment based on comparisons between male read depth and two female HiFi genomes (Female-1: ~1000 kb, Female-2: ~1800 kb). (*) indicate the genes used in this study.

| **CNV Results**  **Female 1** | | | | | | |
| --- | --- | --- | --- | --- | --- | --- |
| Chromosome | Gene | Start | End | Windows | Fold Change | Enriched in |
| Chr04* | ISCGN121770***** | 224454000 | 224468000 | 14 | 3.7091 | female |
| ChrX | ISCGN302940 | 39780221 | 39913323 | 3 | 2.9562 | female |
| Chr12 | ISCGN279270.1 | 84182973 | 84321307 | 5 | 0.2280 | male |
| Chr12 | ISCGN279350.1 | 84709374 | 84780303 | 12 | 0.2892 | male |
| Chr01 | ISCGN013600.1 | 164356965 | 164418915 | 19 | 0.2390 | male |
| Chr12 | ISCGN271940.1 | 28513034 | 28554606 | 3 | 0.1949 | male |
| **Female 2** | | | | | | |
| Chr04 | ISCGN121770 | 224450273 | 224504838 | 14 | 3.4080 | female |
| ChrX* | ISCGN302940***** | 39780221 | 39913323 | 6 | 2.2527 | female |
| Chr01 | ISCGN024100.1 | 260945948 | 260949532 | 8 | 2.5962 | female |
| Chr12 | ISCGN279360.1 | 84794513 | 84810651 | 4 | 3.1616 | female |
| Chr12 | ISCGN279270.1 | 84182973 | 84321307 | 3 | 1.4128 | female |
| Chr12 | ISCGN279350.1 | 84709374 | 84780303 | 6 | 2.3152 | female |
| Chr01 | ISCGN013600.1 | 164356965 | 164418915 | 3 | 0.4295 | male |
| Chr12 | ISCGN271940.1 | 28513034 | 28554606 | 4 | 0.4310 | male |

**Table S2**

**Table S2:** Chromosome, gene ID and location based on genome assembly BIME_Rmic_1.3: GCF_013339725.1, and mean fold change of copy number of genes in adult females relative to male *R. microplus*. Several genes on chromosomes 2 and 8 show higher copy number in females than males. (*) indicate the genes used to sex nymphs in this study (*RAPGEF2* and *NPD*). (**) indicates *EF-1α* which was used as a housekeeping gene.

| **Chromosome** | **Gene** | **Start** | **End** | **Mean fold change of copy number in females** |
| --- | --- | --- | --- | --- |
| Chr1 | LOC119186484 | 247321107 | 247324096 | 0.91 |
|  | LOC119186442 | 247053642 | 247076238 | 1.06 |
| Chr2 | LOC119161905 | 83176545 | 83209732 | 1.92 |
|  | LOC119161173 | 124487303 | 124490459 | 1.34 |
|  | LOC119161094* | 101655155 | 102248900 | 1.80 |
| Chr3 | LOC119164383 | 192887113 | 192890725 | 0.99 |
|  | LOC119163128 | 57919511 | 58461070 | 0.89 |
| Chr4 | LOC119167579 | 150246833 | 150252305 | 1.10 |
|  | LOC119167162 | 73155524 | 73162338 | 1.08 |
| Chr5 | LOC119170533** | 70670053 | 70672667 | 1.00 |
|  | LOC119169704 | 96149227 | 96187497 | 0.99 |
| Chr6 | LOC119172232 | 84904124 | 84907889 | 1.13 |
|  | LOC119171436 | 85082314 | 85083740 | 1.39 |
| Chr7 | LOC119173943 | 147745942 | 147747636 | 1.24 |
|  | LOC119174503 | 120647209 | 120658108 | 1.04 |
| Chr8 | LOC119177190 | 146803630 | 146826965 | 1.78 |
|  | LOC119175720 | 20895823 | 20914529 | 1.57 |
|  | LOC119176070* | 58186459 | 58857799 | 2.11 |
| Chr9 | LOC119178352 | 63397086 | 63441064 | 1.13 |
|  | LOC119177784 | 124353430 | 125031430 | 1.01 |
| Chr10 | LOC119179109 | 56502232 | 56870000 | 0.99 |
|  | LOC119179303 | 111264286 | 111525265 | 1.09 |
| Chr11 | LOC119180762 | 74526927 | 74559738 | 1.09 |
|  | LOC119181641 | 69500151 | 69500714 | 0.91 |

**Table S3.** Descriptive statistics and classification thresholds for SYBR-based and probe-based qPCR assays used to determine sex in adult I. scapularis. Relative copy number ratios were calculated using **2^−dCt** with ***EF-1α*** as the reference gene. Classification cutoffs were derived from the distribution of adult male and female control samples.

| **Statistic** | **SYBR qPCR NPD Male** | **SYBR qPCR NPD Female** | **SYBR qPCR RAPGEF2 Male** | **SYBR qPCR RAPGEF2 Female** | **Probe qPCR Male** | **Probe qPCR Female** | **ddPCR Male** | **ddPCR Female** |
| --- | --- | --- | --- | --- | --- | --- | --- | --- |
| **n** | 13 | 13 | 11 | 11 | 6 | 4 | 4 | 4 |
| **Mean** | 0.545 | 2.019 | 1.390 | 1.965 | 1.085 | 3.741 | 0.493 | 1.228 |
| **SD** | 0.143 | 0.723 | 0.315 | 0.241 | 0.324 | 1.810 | 0.081 | 0.135 |
| **SEM** | 0.040 | 0.201 | 0.095 | 0.073 | 0.132 | 0.905 | 0.040 | 0.067 |
| **Median** | 0.572 | 1.818 | 1.405 | 2.086 | 1.085 | 3.680 | 0.497 | 1.255 |
| **Minimum** | 0.164 | 1.213 | 0.723 | 1.624 | 0.640 | 1.750 | 0.400 | 1.040 |
| **Maximum** | 0.721 | 3.404 | 1.757 | 2.201 | 1.590 | 5.860 | 0.577 | 1.360 |
| **95% CI** | 0.467–0.623 | 1.626–2.412 | 1.204–1.576 | 1.823–2.107 | 0.745–1.425 | 0.860–6.622 | 0.364–0.621 | 1.013–1.442 |
| **Classification cutoff** | < 0.97 | ≥ 0.97 | — | — | < 1.66 | ≥ 1.66 | < 0.60 | ≥ 1.00 |
